# Inflammation and RNA-Related Polymorphisms in Resected Cholangiocarcinoma: Prognostic Associations in Intrahepatic and Perihilar Tumors

**DOI:** 10.1007/s12029-026-01520-z

**Published:** 2026-07-08

**Authors:** Guanwu Wang, Dong Liu, Tarick M. Al-Masri, Smiths S. Lueong, Jens T. Siveke, Tom Luedde, Florian W. R. Vondran, Georg Lurje, Ulf P. Neumann, Lara R. Heij, Jan Bednarsch

**Affiliations:** 1 Department of Surgery and Transplantation, University Hospital RWTH Aachen, Aachen, Germany; 2 Department of Hepatobiliary Surgery, Hunan Provincial People’s Hospital (The First Affiliated Hospital of Hunan Normal University), Changsha, China; 3 Department of Gastrointestinal Surgery, The First Affiliated Hospital of Chongqing Medical University, Chongqing, China; 4 University of Applied Science Aachen, Aachen, Germany; 5 Partner site Essen, Cancer Research Center (DKFZ), German Cancer Consortium (DKTK), University Hospital Essen, Essen, Germany; 6 Bridge Institute of Experimental Tumor Therapy (BIT), Division of Solid Tumor Translational Oncology (DKTK), West German Cancer Center, University Hospital Essen, University of Duisburg-Essen, Essen, Germany; 7 Department of Gastroenterology, Hepatology and Infectious Diseases, Heinrich Heine University Duesseldorf, Duesseldorf, Germany; 8 Department of Surgery and Transplantation, University Hospital Heidelberg, Heidelberg, Germany; 9 Department of Surgery and Transplantation, University Hospital Essen, Essen, Germany; 10 Department of Surgery, Maastricht University Medical Centre (MUMC), Maastricht, Netherlands; 11 Institute of Pathology, University Hospital Essen, Essen, Germany

**Keywords:** Cholangiocarcinoma, Single nucleotide polymorphism, Inflammatory genes, RNA-related genes, Prognosis

## Abstract

**Background:**

Cholangiocarcinoma (CCA) is a heterogeneous and aggressive malignancy of the biliary tract, often classified into intrahepatic (iCCA) and perihilar (pCCA) subtypes. Inflammatory and RNA-regulatory pathways are implicated in CCA pathogenesis. However, the prognostic significance of related single nucleotide polymorphisms (SNPs) remains unclear.

**Method:**

We retrospectively analyzed nine candidate SNPs in inflammation- and RNA-related genes (IL4 rs2243250, IL17A rs4711998, TNIP1 rs7708392, CD274 rs822336, CDKN2B-AS1 rs10965215, NSRP1 rs6505162, MEG3 rs7158663, LOC105370003 rs7315438 and LncRNA PTCSC3 rs944289) in 229 patients with resected CCA (112 iCCA and 117 pCCA). Genotyping was performed using TaqMan assays. Associations between SNP genotypes and recurrence-free survival (RFS), cancer-specific survival (CSS), and overall survival (OS) were evaluated using Kaplan–Meier and Cox regression analyses stratified by anatomical subtype.

**Results:**

The study cohort comprised 229 patients undergoing curative-intent resection for CCA, including 112 iCCA and 117 pCCA cases. Median follow-up was 63 months for iCCA and 89 months for pCCA. Tumor recurrence occurred in 68.8% of iCCA and 54.7% of pCCA patients. No significant associations between SNP genotypes and clinical outcomes were identified in the pCCA cohort. In iCCA, isolated associations with overall survival were observed for TNIP1 rs7708392 and NSRP1 rs6505162, but these findings were not consistently supported across analyses and were limited by small subgroup sizes. No SNP demonstrated consistent associations with recurrence-free survival or cancer-specific survival.

**Conclusion:**

The investigated inflammation- and RNA-related SNPs showed no clinically relevant prognostic value in resected cholangiocarcinoma. Independent validation in larger multicenter cohorts remains warranted.

**Supplementary Information:**

The online version contains supplementary material available at 10.1007/s12029-026-01520-z.

## Introduction

Cholangiocarcinoma (CCA) is a highly aggressive malignancy arising from biliary epithelium, comprising about 10–20% of primary liver cancers and 3% of gastrointestinal cancers [1]. Anatomically, CCA is classified into intrahepatic (iCCA), perihilar (pCCA, Klatskin tumor), and distal subtypes [2]. Perihilar tumors are the most common (50–60% of cases), followed by distal (30%) and intrahepatic (10%) [3]. Although the incidence is relatively low in Western countries (0.3–6 per 100,000), it has been rising globally and is markedly higher in parts of Southeast Asia (7–15 per 100,000) [4]. CCA predominantly affects older adults, with a male predominance partly due to risk factors such as primary sclerosing cholangitis, hepatolithiasis, liver fluke infection, viral hepatitis, obesity, and diabetes [5].

Long-term survival remains sparse in patients with CCA. Recent advances in systemic therapy have shown some promise in improving survival figures: for instance, pemigatinib (a selective FGFR2 inhibitor) has achieved objective responses in FGFR2-fusion positive iCCA and adding the immune checkpoint inhibitor durvalumab to first-line chemotherapy (TOPAZ-1 trial) modestly improved survival [6–8]. Today, surgical resection offers the only potential cure and overall best oncological outcome among all therapeutic approaches, but merely 25% of patients are eligible at diagnosis because of advanced disease stage [9]. However, surgery in case of CCA commonly refers to major liver resection and eventually vascular resection and/or biliary reconstruction resulting in notable postoperative morbidity and mortality. Also, even after curative-intent surgery, recurrence is common (50–70%) and diminishing survival propabilites [10]. Thus identifying patients which profit most from invasive surgery on the basis of easily assessable biomarkers is of major interest for distinct patient selection.

Single nucleotide polymorphisms (SNPs) are the most common form of human genetic variation, occurring roughly once per 1000 base pairs [11]. Certain SNPs can alter gene function or expression and have been linked to disease susceptibility, treatment response, and clinical outcomes [12]. In cancer, SNPs may influence key pathways (e.g. DNA repair, inflammation) that drive tumor progression and therapy resistance [13]. Chronic inflammation, in particular, plays a critical role in cholangiocarcinogenesis [14]. Genetic polymorphisms in inflammatory regulators can modulate cytokine production and tumor microenvironment, thereby impacting cancer development and prognosis [15].

Our group has recently reported the prognostic value of DNA-related SNPs for cholangiocarcinoma with positive associations [16, 17]. Further, genetic variations, particularly SNPs within inflammatory and RNA-regulatory pathways, have been increasingly recognized as potential modifiers of cancer progression and patient prognosis. However, the role of these specific SNPs in cholangiocarcinoma (CCA), and their possible differences between intrahepatic and perihilar tumor subtypes, remains unclear. Therefore, we selected candidate SNPs which showed biological significance and prognostic value in other entities and aimed to assess whether these selected SNPs in inflammation and RNA regulation-related genes might serve as prognostic biomarkers for patients undergoing surgical resection for CCA (supplementary table S1).

## Materials and Methods

### Study Cohort

Between May 2009 and December 2020, a total of 229 consecutive patients underwent curative-intent surgical resection for either intrahepatic (iCCA) or perihilar cholangiocarcinoma (pCCA) at RWTH Aachen University Hospital. Only individuals discharged from the hospital and with no 90-day mortality were considered for analysis (117 pCCA, 112 iCCA). All participants were followed up postoperatively. Informed consent was obtained from the patients. The study protocol received approval from the institutional review board of RWTH Aachen University (EK 252/15, EK 206/09) and was conducted in accordance with the Declaration of Helsinki and Good Clinical Practice standards.

### DNA Isolation and Quantification

For each patient, non-tumorous liver tissue was retrieved from formalin-fixed, paraffin-embedded (FFPE) blocks archived at RWTH Aachen University Hospital. A single pathologist reviewed hematoxylin and eosin–stained sections to confirm tissue suitability. DNA was extracted from 10 μm FFPE sections using the QIAamp DNA FFPE Tissue Kit (Qiagen, Hilden, Germany), strictly following the manufacturer’s instructions. Briefly, sections were deparaffinised with 1 mL xylene (vortexed for 2 min at room temperature) and washed in ethanol to remove solvent residues. Protein digestion was performed with 20 µL proteinase K and 180 µL ATL buffer at 56 °C for 1 h, followed by heat inactivation at 90 °C for 1 h. The lysate was applied to a silica spin column, washed twice, and dried before elution in 50 µL elution buffer. DNA concentration was measured using a BioTek Synergy HT spectrophotometer (USA), and all samples were stored at − 20 °C. Working solutions were adjusted to 10 ng/µL.

### Selection of SNPs

Single nucleotide polymorphisms (SNPs) in inflammation- and RNA-related genes were chosen according to three predefined criteria: (i) known functional relevance, (ii) a minor allele frequency high enough to enable reliable statistical analysis, and (iii) absence of prior associations with cholangiocarcinoma in the literature. Based on these requirements the following nine SNPs were analyzed: IL4 (Interleukin 4) rs2243250, IL17A (Interleukin 17 A) rs4711998, TNIP1 (TNFAIP3 Interacting Protein 1) rs7708392, CD274 (CD274 Molecule) rs822336, CDKN2B-AS1 (Cyclin-dependent kinase inhibitor 2B antisense RNA 1) rs10965215, NSRP1 (Nuclear Speckle Splicing Regulatory Protein 1) rs6505162, MEG3 (Maternally Expressed Gene 3) rs7158663, LOC105370003 (Predicted long non-coding RNA LOC105370003) rs7315438, and the lncRNA PTCSC3 (Papillary Thyroid Carcinoma Susceptibility Candidate 3) rs944289. (details provided in Supplementary Table S1).

### Genotyping Procedures

Genotyping was carried out using TaqMan SNP Genotyping Assays (Thermo Fisher Scientific, CA, USA) on an ABI 7500 Real-Time PCR platform. Each reaction (total volume 5.05 µL) consisted of 2.5 µL TaqMan Genotyping Master Mix, 0.25 µL of the 40× probe mix, 1.0 µL nuclease-free water, and 1.3 µL genomic DNA (~ 10 ng). PCR cycling involved an initial activation step at 95 °C for 10 min, followed by 40 cycles of denaturation at 95 °C for 15 s and annealing/extension at 60 °C for 60 s. To ensure assay reproducibility, approximately 5% of genotypes were retested, and an additional 10% of samples, including positive and negative controls, were re-analysed for quality control. Allelic discrimination plots (VIC dye for allele 1; FAM dye for allele 2) were reviewed for cluster separation and genotype assignment, showing clear grouping for homozygotes, heterozygotes, and no-template controls.

### Statistical Analysis

Each SNP was tested for Hardy–Weinberg equilibrium using Pearson’s χ² test, with *p* > 0.05 indicating equilibrium. In case of deviations from the expected genotype distributions, the SNPs were not included in further analysis. Outcome analysis focused on three endpoints: recurrence-free survival (RFS), cancer-specific survival (CSS), and overall survival (OS). RFS was defined as the interval between surgery and first documented recurrence. CSS was defined as the time from surgery to death from cholangiocarcinoma, censoring deaths from unrelated causes. OS was defined as time from resection to death from any cause; no perioperative deaths occurred. Categorical data were analysed using Fisher’s exact test, and continuous variables using appropriate non-parametric methods. Survival distributions were estimated via the Kaplan–Meier method and compared using the log-rank test. Cox proportional hazards models were applied to evaluate the impact of variables on RFS, CSS, and OS, yielding hazard ratios (HR) with corresponding 95% confidence intervals (CI). Variables with *p* < 0.1 in univariate analysis were entered into multivariable models. All analyses were performed in IBM SPSS Statistics version 26 (IBM Corp., Armonk, NY, USA), with two-sided p-values < 0.05 considered statistically significant.

## Results

### Characteristics of the Study Cohort

A total of 229 patients who underwent curative-intent resection for cholangiocarcinoma at RWTH Aachen University Hospital between 2009 and 2020 were included in the final analysis. Of these, 112 (48.9%) had intrahepatic cholangiocarcinoma (iCCA) and 117 (51.1%) had perihilar cholangiocarcinoma (pCCA) (Table 1). Compared to pCCA patients, those with iCCA were more often female (56.3% vs. 33.3%) and slightly younger (median age 65 vs. 68 years). Hemihepatectomy, extended hemihepatectomy and trisectionectomy were the most common procedures in both groups. The R0 resection rate was similar (86.6% in iCCA vs. 82.9% in pCCA), but nodal involvement was more frequent in pCCA (42.7% vs. 30.4%). Postoperative complications ≥ Clavien–Dindo grade IIIa were more frequent in pCCA (55.5%) than in iCCA (36.6%). Median follow-up time was 63 months in iCCA and 89 months in pCCA. Recurrence occurred in 68.8% of iCCA patients and 54.7% of pCCA patients. More details are displayed in Table 1.

**Table 1:** Patient characteristics

Variables	iCCA(*n* = 112)	pCCA(*n* = 117)
**Demographics**		
Gender, M/F (%)	49(43.8)/63(56.3)	78(66.7)39/(33.3)
Age (years)	65(58–75)	69(58–74)
ASA, n (%)		
I	3(2.7)	6(5.1)
II	45(40.2)	47(40.2)
III	59(52.7)	58(49.6)
IV	5(4.5)	6(5.1)
V	0	0
Bismuth type, n (%)		
I	/	7(6.0)
II	/	13(11.1)
IIIa	/	29(24.8)
IIIb	/	33(28.2)
IV	/	35(29.9)
Cholangitis, n (%)	4(3.6)	30(25.6)
Portal vein embolization, n (%)	7(6.3)	41(35.0)
Preoperative Chemotherapy, n (%)	9(8.0)	5(4.3)
**Clinical chemistry**		
AST (U/l)	34(26–47)	47(34–95)
ALT (U/l)	29(20–49)	69(36–144)
Albumin(g/dL)	4.4(4.0-4.6)	3.9(3.5–4.2)
AP, U/L	118(84–215)	254(159–423)
CA19-9(U/ml)	39.5(13.9-236.3)	85.8(31.2-287.7)
CRP (mg/l)	7.5(2.8–19.4)	11.8(5.4–34.7)
GGT (U/l)	113(65–275)	472(216–756)
Hemoglobin (g/dl)	13.1(12.2–14.3)	12.4(11.2–13.3)
INR	1.0(0.9–1.1)	1.0(0.9–1.1)
Platelet count (/nl)	252(202–306)	293(229–388)
Prothrombin time (%)	100(90–108)	96(81–105)
Total bilirubin (mg/dl)	0.5(0.3–0.7)	1.1(0.5–2.8)
**Operative Data**		
Intraoperative PRBC, n(%)	31(72.3)	54(46.2)
Intraoperative FFP, n(%)	38(33.9)	68(58.1)
Operative time (minutes)	295(230–359)	420(356–485)
Operative procedure, n (%)		
Hemihepatectomy	44(39.2)	29(24.8)
Extended hemihepatectomy	17(15.1)	54(46.2)
Trisectionectomy	12(10.8)	24(20.6)
Hepatoduodenoectomy	0(0)	5(4.3)
ALPPS	10(8.9)	1(0.9)
Others	29(25.8)	4(3.4)
Time to surgery (days*)	45(29–83)	38(24–56)
**Pathological examination**		
LVI, n (%)	20(17.9)	26(22.2)
MVI, n (%)	5(4.5)	2(1.7)
R0 resection, n (%)	97(86.6)	97(82.9)
pT category n (%)		
1	46(41.1)	10(8.6)
2	46(41.1)	72(61.5)
3	12(10.7)	26(22.2)
4	8(7.1)	9(7.7)
pN category, n (%)		
N0	69(61.6)	67(57.3)
N1	34(30.4)	50(42.7)
Tumor grading, n (%)		
G1	0	7(6.0)
G2	73(65.2)	83(70.9)
G3	26(23.2)	22(18.8)
G4	3(2.7)	1(0.9)
**Postoperative Data**		
Intensive care, days	1(1–1)	1(1–2)
Hospitalization, days	12(8–22)	17(12–36)
Postoperative complications, n (%)		
No complications	43(38.4)	19(16.2)
Clavien-Dindo I	4(3.6)	8(6.8)
Clavien-Dindo II	24(21.4)	33(28.2)
Clavien-Dindo IIIa	25(22.3)	25(28.2)
Clavien-Dindo IIIb	10(8.9)	19(16.2)
Clavien-Dindo IVa	5(4.5)	8(6.8)
Clavien-Dindo IVb	1(0.9)	5(4.3)
Clavien-Dindo V	0	0
**Oncologic Data**		
Adjuvant chemotherapy, n (%)	42(37.5)	31(26.5)
Recurrence, n (%)	77(68.8)	64(54.7)
Median RFS, months (95% CI)	12(7–17)	37(23–51)
Median CSS, months (95% CI)	28(21–35)	49(24–74)
Median OS, months (95% CI)	25(18–32)	33(20–46)

Data are presented as median and interquartile range unless otherwise indicated. Time to surgery refers to the interval between diagnosis and surgical resection. ALPPS, associating liver partition and portal vein ligation for staged hepatectomy; ALT, alanine aminotransferase; AP, alkaline phosphatase; ASA, American Society of Anesthesiologists; AST, aspartate aminotransferase; BMI, body mass index; CA19-9, carbohydrate antigen 19 − 9; CRP, C-reactive protein; CSS, cancer-specific survival; FFP, fresh frozen plasma; GGT, gamma-glutamyl transferase; INR, international normalized ratio; LVI, lymphovascular invasion; MVI, microvascular invasion; OS, overall survival; PRBC, packed red blood cells; RFS, recurrence-free survival

### Single Nucleotide Polymorphisms in Intrahepatic Cholangiocarcinoma

Genotype data were available for most of the 112 iCCA patients across the nine SNPs analyzed. However, a small number of samples failed genotyping for specific loci: rs2243250 (IL4), rs4711998 (IL17A), rs7708392 (TNIP1), rs6505162 (NSRP1), rs7158663 (MEG3), and rs944289 (PTCSC3) each had one missing case, while rs10965215 (CDKN2B-AS1) and rs7315438 (LOC105370003) had two missing cases, and rs822336 (CD274) had three missing cases. All SNPs were in Hardy–Weinberg equilibrium (*p* > 0.05) except for rs7158663 (*p* = 0.02) and rs944289 (*p* = 0.03), which showed slight deviations from expected genotype distributions and were therefore not included in further analysis (Supplementary Table S3).

### Kaplan–Meier Analysis in Intrahepatic Cholangiocarcinoma

**Fig. 1:**
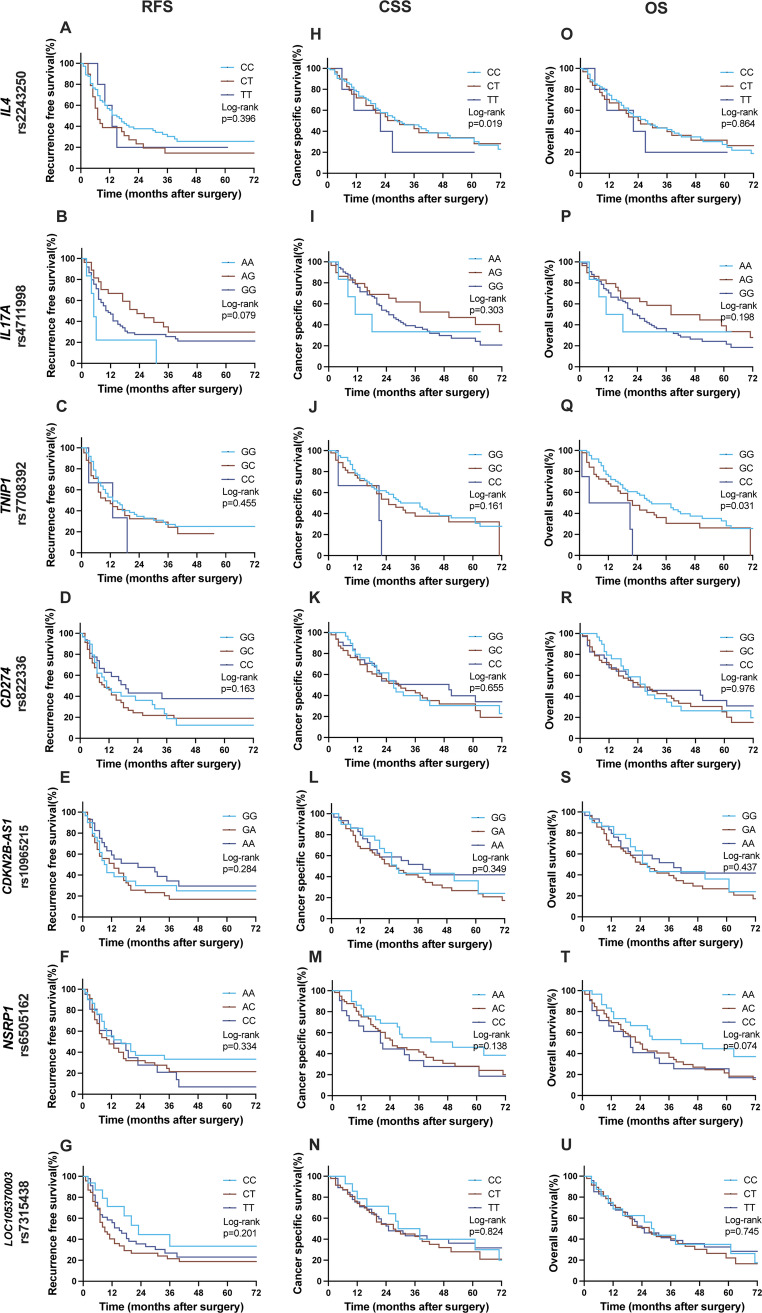
Kaplan-Meier survival analysis of candidate SNPs in patients with intrahepatic cholangiocarcinoma. Recurrence-free survival, cancer-specific survival and overall survival stratified by genotype for SNPs IL4 rs2243250, IL17A rs4711998, TNIP1 rs7708392, CD274 rs822336, CDKN2B-AS1 rs10965215, NSRP1 rs6505162 and LOC105370003 rs7315438. Survival differences were analyzed by the log-rank test. Significant survival differences (*p* < 0.05) are highlighted. CD274, CD274 Molecule; CDKN2B-AS1, Cyclin-dependent kinase inhibitor 2B antisense RNA 1; IL4, Interleukin 4; IL17A, Interleukin 17 A; LOC105370003, Predicted long non-coding RNA LOC105370003; MNSRP1, Nuclear Speckle Splicing Regulatory Protein 1; TNIP1, TNFAIP3 Interacting Protein 1

Kaplan–Meier survival analysis revealed significant genotype-based differences for two SNPs. The detailed Kaplan–Meier survival curves for RFS, CSS, and OS stratified by SNP genotypes in iCCA patients are presented in Fig. 1. Among patients with iCCA, those carrying the TNIP1 rs7708392 CC genotype exhibited significantly reduced OS compared to individuals with GG or GC genotypes (median OS 4.0 vs. 27.0 months; log-rank *p* = 0.020). A similar, though not statistically significant, trend was observed for cancer-specific survival, with CC genotype carriers having a median CSS of 21.0 months versus 29.0 months in the GG/GC group (*p* = 0.078) (Table 2; Fig. 1). However, due to the very small number of CC genotype cases (*n* = 4), this association should be interpreted cautiously. It may represent an incidental finding rather than a meaningful biological association, especially given the absence of significant associations with RFS or CSS, which are clinically more relevant endpoints.

**Table 2:** Single nucleotide polymorphism frequencies and associations with recurrence-free survival, cancer-specific survival and overall survival in intrahepatic cholangiocarcinoma

SNP	*N* (%)	Recurrence-free survival					Cancer-specific survival				Overall survival			
		Median (95% CI)	*p* value*		HR (95% CI)	*p* value^#^	Median (95% CI)	*p* value*	HR (95% CI)	*p* value^#^	Median (95% CI)	*p* value*	HR (95% CI)	*p* value^#^
**Recessive model**														
rs2243250				0.948				0.453				0.642		
CC/CT	107(95.5)	12(6.2–17.8)			1		29(19.2–38.8)		1		25(16.8–33.2)		1	
TT	5(4.5)	13(6.6–19.4)			0.968(0.353–2.654)	0.949	22(0-45.6)		1.464(0.532–4.026)	0.460	22(0-45.6)		1.266(0.462–3.467)	0.647
rs4711998				0.358				0.211				0.178		
AA/AG	37(33.0)	20(7.0-32.9)			1		38(6.3–69.7)		1		38(7.9–68.0)		1	
GG	75(67.0)	10.0(6.6–13.4)			1.250(0.768–2.033)	0.369	25(18.5–31.5)		1.388(0.824–2.338)	0.218	24(17.5–30.5)		1.384(0.856–2.236)	0.185
rs7708392				0.408				0.078				**0.020**		
GG/GC	108(96.4)	12(7.0–17.0)			1		29(19.7–38.3)		1		27(18.5–35.4)		1	
CC	4(3.6)	13(0–29.0)			1.605(0.503–5.120)	0.424	21(0-48.2)		2.716(0.841–8.772)	0.095	4(0-23.6)		3.125(1.126–8.677)	**0.029**
rs822336				0.059				0.385				0.850		
GG/GC	77(68.7)	10(5.5–14.5)			1		27(20.6–33.4)		1		25(17.5–32.5)		1	
CC	35(31.3)	19(12.6–23.4)			0.605(0.352–1.038)	0.068	50(23.2–76.8)		0.790(0.462–1.353)	0.391	22(10.7–33.3)		0.955(0.592–1.542)	0.851
rs10965215				0.158				0.258				0.205		
GG/GA	82(73.2)	10(5.6–14.4)			1		27(19.6–34.4)		1		25(19.2–30.8)		1	
AA	30(26.8)	16(0-37.7)			0.694(0.412–1.169)	0.170	38(21.4–54.6)		0.728(0.416–1.272)	0.264	32(9.0-54.9)		0.716(0.423–1.210)	0.212
rs6505162				0.602				0.203				0.202		
AA/AC	89(79.5)	11(6.7–15.3)			1		29(18.1–39.9)		1		27(19.6–34.2)		1	
CC	23(20.5)	13.0(0-26.1)			1.154(0.665–2.006)	0.610	21(10.3–31.7)		1.441(0.813–2.553)	0.210	20(9.9–30.1)		1.411(0.825–2.413)	0.209
rs7315438				0.861				0.700				0.443		
CC/CT	65(58.0)	11(6.5–15.5)			1		28(16.5–39.5)		1		27(19.2–34.8)		1	
TT	47(42.0)	13(5.8–20.2)			0.961(0.608–1.519)	0.864	25(14.9–35.0)		0.911(0.564–1.471)	0.703	25(14.1–35.9)		0.841(0.538–1.315)	0.449
**Co-dominant model**														
rs2243250				0.396				**0.019**				0.864		
CC	76 (67.9%)	13(7.3–18.7)			1		42(26.5–57.5)		1		27(17.6–36.4)		1	
CT	31 (27.7%)	7(4.5–9.5)			1.399(0.850–2.301)	0.186	20(14.1–25.9)		1.009(0.592–1.720)	0.974	25(10.6–39.4)		0.933(0.568–1.533)	0.784
TT	5 (4.5%)	13(6.5–19.4)			1.064(0.383–2.955)	0.905	50(37.8–62.2)		1.468(0.528–4.083)	0.462	22(0-45.6)		1.241(0.449–3.432)	0.677
rs4711998				0.079				0.303				0.198		
AA	7 (6.3%)	5(3.0–7.0)			1		11(0–23.0)		1		11(3.3–18.7)		1	
AG	30 (26.8%)	20(8.9–31.0)			0.349(0.129–0.946)	**0.039**	50(19.3–80.7)		0.574(0.191–1.721)	0.322	38(8.0–68.0)		0.520(0.193–1.401)	0.196
GG	75 (67.0%)	10(6.6–13.4)			0.507(0.201–1.278)	0.150	25(18.5–31.8)		0.871(0.314–2.417)	0.791	24(17.5–30.5)		0.802(0.321–2.003)	0.636
rs7708392				0.455				0.161				**0.031**		
GG	63 (56.3%)	13(7.5–18.5)			1		38(23.8–52.1)		1		30(16.3–43.7)		1	
GC	45 (40.2%)	8(0.9–15.1)			1.248(0.782–1.991)	0.353	25(14.0–36.0)		1.206(0.737–2.974)	0.456	22(14.6–29.4)		1.332(0.839–2.115)	0.225
CC	4 (3.6%)	13(0–29.0)			1.751(0.540–5.678)	0.351	21(0-48.2)		2.936(0.891–9.672)	0.077	4(0-23.6)		3.534(1.245–10.035)	**0.018**
rs822336				0.163				0.655				0.976		
GG	29 (25.9%)	11(6.1–15.9)			1		27(18.8–35.2)		1		27(17.8–36.2)		1	
GC	48 (42.9%)	9(2.7–15.3)			1.062(0.629–1.793)	0.822	29(16.0–42.0)		1.087(0.621–1.904)	0.770	25(11.7–38.4)		1.031(0.608–1.747)	0.911
CC	35 (31.3%)	19(12.6–25.4)			0.627(0.335–1.174)	0.145	50(23.2–76.8)		0.831(0.439–1.573)	0.570	22(10.7–33.3)		0.972(0.548–1.726)	0.924
rs10965215				0.284				0.349				0.437		
GG	32 (28.6%)	10(7.1–12.9)			1		27(21.1–32.9)		1		25(18.3–31.7)		1	
GA	50 (44.6%)	11(3.4–18.6)			1.205(0.699–2.079)	0.503	27(16.8–37.2)		1.288(0.725–2.291)	0.388	24(14.4–33.6)		0.947(0.969–1.577)	0.834
AA	30 (26.8%)	16(0-37.7)			0.780(0.416–1.463)	0.439	38(21.4–54.6)		0.860(0.434–1.703)	0.665	32(9.0–55.0)		0.692(0.375–1.276)	0.238
rs6505162				0.334				0.138				0.074		
AA	30 (26.8%)	16(3.6–28.4)			1		50(11.2–88.8)		1		41(8.1–73.9)		1	
AC	59 (49.6%)	10(4.1–15.9)			1.463(0.848–2.526)	0.172	25(16.3–33.7)		1.589(0.886–2.848)	0.120	24(18.6–29.4)		1.699(0.985–2.930)	0.057
CC	23 (20.5%)	13(0-26.107			1.475(0.757–2.875)	0.254	21(10.3–31.7)		1.957(0.966–3.966)	0.062	20(9.6–30.1)		2.012(1.033–3.919)	**0.040**
rs7315438				0.201				0.824				0.745		
CC	16 (14.3%)	23(13.5–32.5)			1		29(13.7–44.3)		1		28(20.2–35.8)		1	
CT	49 (43.8%)	9(6.6–11.4)			1.940(0.903–4.170)	0.090	27(10.1–43.9)		1.188(0.582–2.423)	0.636	27(18.3–35.7)		1.009(0.541–1.882)	0.978
TT	47 (42.0%)	13(5.8–20.2)			1.597(0.734–3.475)	0.238	25(15.0–35.0)		1.035(0.503–2.133)	0.925	25(14.1–35.9)		0.847(0.452–1.588)	0.604
**Dominant model**														
rs2243250				0.216				0.797				0.900		
CC	76 (67.9%)	13(7.3–18.7)			1		49(19.0–79.0)		1		27(17.6–36.4)		1	
CT/TT	36 (32.2%)	8(4.9–11.1)			1.336(0.834–2.141)	0.228	51(13.6–88.4)		1.067(0.648–1.758)	0.799	24(14.8–33.2)		0.971(0.608–1.550)	0.901
rs4711998				0.076				0.617				0.860		
AA	7 (6.3%)	5(3.0–7.0)			1		11(0–23.0)		1		31(10.6–51.4)		1	
AG/GG	105 (93.8%)	13(8.4–17.6)			0.454(0.182–1.136)	0.091	28(18.7–37.3)		0.775(0.282–2.130)	0.621	38(23.6–52.4)		0.711(0.287–1.761)	0.460
rs7708392				0.274				0.310				0.890		
GG	63 (56.3%)	13(7.5–18.5)			1		38(23.9–52.1)		1		38(27.1–48.9)		1	
GC/CC	49 (43.8%)	10(4.1–15.9)			1.281(0.813–2.018)	0.286	22(14.7–29.3)		1.278(0.792–2.062)	0.316	31(8.7–53.3)		1.425(0.910–2.231)	0.121
rs822336				0.564				0.934				0.489		
GG	29 (25.9%)	11(6.1–15.9)			1		27(18.8–35.2)		1		32(12.3–51.7)		1	
GC/CC	83 (74.2%)	13(7.1–18.9)			0.869(0.532–1.418)	0.573	29(13.1–44.9)		0.978(0.582–1.645)	0.934	33(16.9–49.1)		1.006(0.621–1.630)	0.980
rs10965215				0.948				0.683				0.984		
GG	32 (28.6%)	10(7.1–12.9)			1		27(21.1–32.9)		1		27(0-59.7)		1	
GA/AA	79 (70.5%)	13(7.7–18.3)			1.017(0.610–1.696)	0.949	29(16.4–41.6)		1.119(0.647–1.935)	0.686	33(23.2–42.8)		0.848(0.526–1.368)	0.499
rs6505162				0.139				0.065				0.860		
AA	30 (26.8%)	16(3.6–28.4)			1		50(11.2–88.8)		1		38(13.6–62.4)		1	
AC/CC	82 (70.1%)	11(5.8–16.2)			1.467(0.871–2.471)	0.150	25(16.8–33.2)		1.676(0.958–2.933)	0.071	32(19.0–45.0)		1.771(1.048–2.994)	**0.033**
rs7315438				0.115				0.757				0.461		
CC	16 (14.3%)	23(13.5–32.5)			1		29(13.7–44.3)		1		49(23.8–74.2)		1	
CT/TT	96 (85.8%)	10(6.2–13.8)			1.766(0.848–3.677)	0.128	27(18.7–35.3)		1.111(0.568–2.172)	0.759	33(22.7–43.3)		0.923(0.518–1.645)	0.786

The following SNPs were analyzed: IL4 rs2243250, IL17A rs4711998, TNIP1 rs7708392, CD274 rs822336, CDKN2B-AS1 rs10965215, NSRP1 rs6505162 and LOC105370003 rs7315438. CD274, CD274 Molecule; CDKN2B-AS1, Cyclin-dependentkinase inhibitor 2B antisense RNA 1; IL4, Interleukin 4; IL17A, Interleukin 17 A; LOC105370003, Predicted long non-coding RNA LOC105370003; NSRP1, Nuclear Speckle Splicing Regulatory Protein 1; TNIP1, TNFAIP3 Interacting Protein1. ^*^, Kaplan–Meier survival analysis;^#^, univariate Cox regression analyses

Although the univariate log-rank analysis for NSRP1 rs6505162 did not reach statistical significance (*p* = 0.074 for overall survival), patients with the CC genotype demonstrated a numerically shorter median OS of 20.0 months compared to those with the AC genotype (24.0 months) and AA genotype (41.0 months) (Table 2; Fig. 1E).

For the remaining SNPs (e.g. IL4 rs2243250, IL17A rs4711998), no significant differences in RFS, CSS or OS were observed across genotypes (Table 2; Fig. 1).

### Associations Between Patient Characteristics, Single Nucleotide Polymorphisms and Outcomes in Intrahepatic Cholangiocarcinoma

Univariate Cox regression identified TNIP1 rs7708392 CC genotype as significantly associated with inferior OS (HR = 3.125, 95% CI 1.126–8.677, *p* = 0.029) (Table 2). NSRP1 rs6505162 AC genotype also showed a trend (HR = 1.699, 95% CI 0.985–2.930, *p* = 0.057), although not statistically significant.

In multivariate Cox analysis adjusted for clinical covariates (Table 3), TNIP1 rs7708392 remained an independent predictor of both OS (HR = 141.712, 95% CI 16.668–1204.822, *p* < 0.001) and CSS (HR = 13.532, 95% CI 2.191–83.571, *p* = 0.005). Similarly, NSRP1 rs6505162 AC genotype was associated with reduced OS (HR = 3.486, 95% CI 1.465–8.295, *p* = 0.005). However, the very small number of CC genotype cases (*n* = 4) should be kept in mind by interpreting the results. No SNPs were significantly associated with recurrence-free survival (RFS) in multivariate models. A complete univariate analysis including all clinical variables and SNP data is presented in supplementary table S5.

**Table 3:** Multivariable Cox regression analysis of recurrence-free, cancer-specific and overall survival in intrahepatic cholangiocarcinoma

Variables	Recurrence-free survival		Cancer-specific survival		Overall survival	
	HR (95% CI)	*p* value	HR (95% CI)	*p* value	HR (95% CI)	*p* value
Neoadjuvant therapy (No = 1)	3.139(1.088–9.062)	0.034	----	--	----	--
Intraoperative PRBC (No = 1)	----	--	2.552(1.283–5.077)	0.008	2.908(1.452–5.824)	0.003
LVI (No = 1)	3.563(1.851–6.857)	<0.001	----	--	----	--
Tumor stage UICC (I/II = 1)	----	--	2.065(0.009–0.469)	0.007	0.078(0.013–0.486)	0.006
pT category (pT1-2 = 1)	----	--	5.382(1.612–17.968)	0.006	11.530(3.082–43.136)	<0.001
N category (pN0 = 1)	----	--	39.371(6.266-247.388)	<0.001	32.428(5.402-194.662)	<0.001
rs6505162						
AA	----	--	----	--	1	
AC	----	--	----	--	3.486(1.465–8.295)	0.005
CC	----	--	----	--	0.878(0.256–3.011)	0.837
rs7708392						
GG	----	--	1		1	
GC	----	--	0.998(0.513–1.939)	0.995	1.407(0.676–2.928)	0.361
CC	----	--	13.532(2.191–83.571)	0.005	141.712 (16.668-1204.822)	<0.001

Only significant parameters are shown. LVI, lymph vascular invasion; PRBC, packed red blood cells

### Single Nucleotide Polymorphisms in Perihilar Cholangiocarcinoma

Genotyping data for the nine investigated SNPs were available for most of the 117 patients with perihilar cholangiocarcinoma (pCCA). However, a small number of samples were not successfully genotyped at specific loci: rs2243250 (*IL4*) had one missing case, rs822336 (*CD274*) had one missing, rs10965215 (*CDKN2B-AS1*) and rs7315438 (*LOC105370003*) each had one missing case, while rs7158663 (*MEG3*) and rs944289 (*PTCSC3*) each had two missing genotypes. Most SNPs were in Hardy–Weinberg equilibrium (HWE, *p* > 0.05), except for rs2243250 (*p* = 0.01), rs7158663 (*p* = 0.04), and rs944289 (*p* = 0.01), which showed slight deviations from expected genotype distributions and were therefore not included in further analysis (Supplementary Table S4).

### Kaplan–Meier Analysis in Perihilar Cholangiocarcinoma

**Fig. 2:**
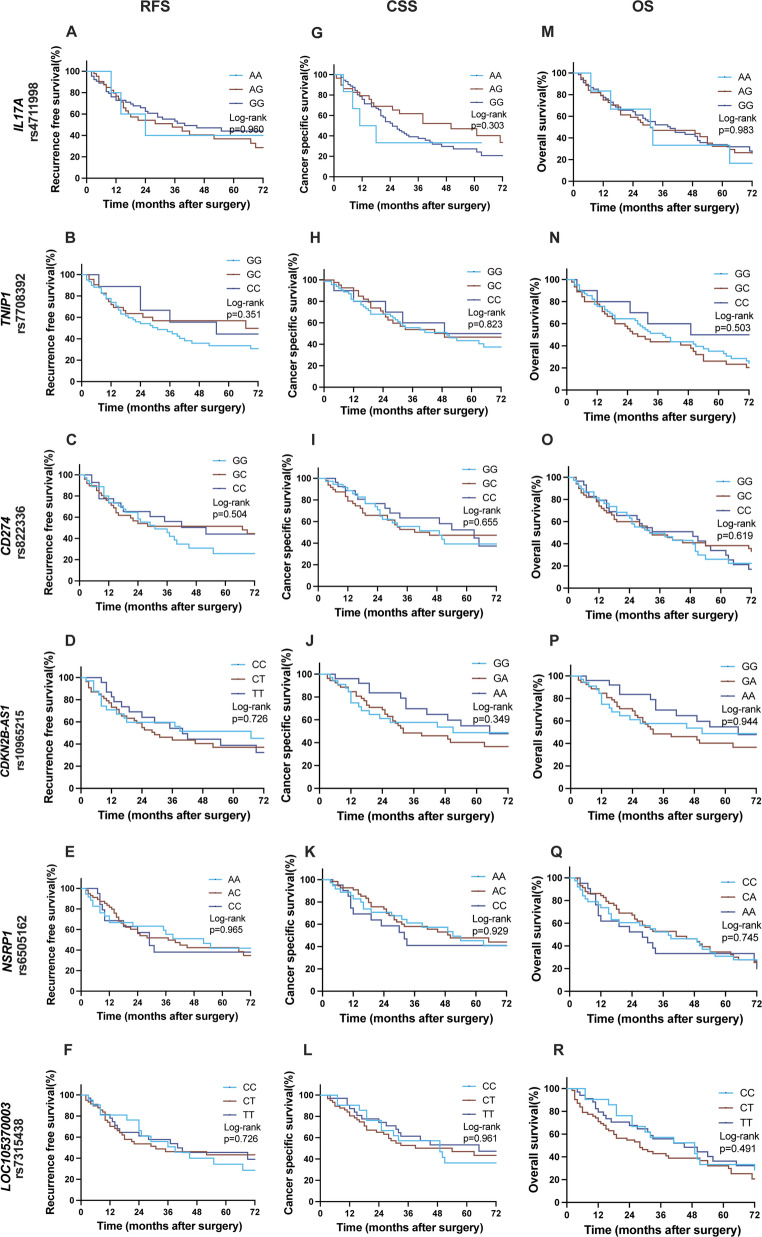
Kaplan-Meier survival analysis of candidate SNPs in patients with perihilar cholangiocarcinoma. Recurrence-free survival, cancer-specific survival and overall survival stratified by genotype for SNPs IL17A rs4711998, TNIP1 rs7708392, CD274 rs822336, CDKN2B-AS1 rs10965215, NSRP1 rs6505162 and LOC105370003 rs7315438. Survival differences were analyzed by the log-rank test. No significant differences were identified. CD274, CD274 Molecule; CDKN2B-AS1, Cyclin-dependent kinase inhibitor 2B antisense RNA 1; IL17A, Interleukin 17 A; LOC105370003, Predicted long non-coding RNA LOC105370003; NSRP1, Nuclear Speckle Splicing Regulatory Protein 1; TNIP1, TNFAIP3 Interacting Protein 1

Kaplan–Meier analysis showed no statistically significant differences in RFS, CSS, or OS for any SNPs (all log-rank *p* > 0.1) (Table 4; Fig. 2). For TNIP1 rs7708392, median overall survival did differ between genotype groups in patients with pCCA, with CC carriers having a median OS of 84.0 months compared to 33.0 months for those with GG or GC genotypes but failed to show statistical significance due to the low number individuals (*p* = 0.318). Similarly, no significant survival differences were observed across genotypes for IL17A rs4711998 and IL4 rs2243250 (Table 4; Fig. 2).

**Table 4:** Single nucleotide polymorphism frequencies and associations with recurrence-free survival, cancer-specific survival and overall survival in perihilar cholangiocarcinoma

SNP	*N* (%)	Recurrence-free survival					Cancer-specific survival				Overall survival			
		Median (95% CI)		*p* value*	HR (95% CI)	*p* value^#^	Median (95% CI)	*p* value*	HR (95% CI)	*p* value^#^	Median (95% CI)	*p* value*	HR (95% CI)	*p* value^#^
**Recessive model**														
rs4711998				0.535				0.928				0.916		
AA/AG	50(42.7)		28(3.6–52.5)		1		51(22.5–79.5)		1		31(7.7–54.3)		1	
GG	67(57.3)		40(13.9–66.1)		0.855(0.520–1.408)	0.539	49(4.8–93.2)		0.977(0.588–1.624)	0.929	39(23.7–54.3)		0.978(0.638–1.497)	0.917
rs7708392				0.426				0.793				0.311		
GG/GC	108(92.3)		37(21.8–52.2)		1		84(0-235.9)		1		33(22.1–43.9)		1	
CC	9(7.7)		55(0-140.9)		0.691(0.275–1.738)	0.433	50(24.0-75.9)		0.892(0.379–2.099)	0.793	84(0-235.9)		0.671(0.306–1.469)	0.318
rs822336				0.536				0.728				0.708		
GG/GC	88(75.2)		31(15.6–46.4)		1		45(25.9–64.1)		1		32(21.1–42.9)		1	
CC	29(24.8)		52(21.4–82.6)		0.830(0.458–1.505)	0.540	63(39.1–86.9)		0.900(0.495–1.637)	0.730	49(21.8–76.2)		1.095(0.678–1.767)	0.711
rs10965215				0.674				0.350				0.750		
GG/GA	90(76.9)		36(16.2–55.8)		1		45(26.2–63.8)		1		29(18.1–39.9)		1	
AA	27(23.1)		40(21.3–58.7)		0.881(0.486–1.598)	0.677	65(36.9–93.1)		0.748(0.405–1.383)	0.355	54(39.5–68.5)		0.925(0.569–1.502)	0.752
rs6505162				0.931				0.711				0.444		
AA/AC	96(82.1)		40(18.2–61.8)		1		51(29.5–72.5)		1		41(25.7–56.3)		1	
CC	21(17.9)		29(19.8–38.2)		0.972(0.507–1.865)	0.932	32(19.0–45.0)		1.131(0.588–2.174)	0.712	28(11.6–44.4)		1.227(0.722–2.087)	0.449
rs7315438				0.976				0.992				0.539		
CC/CT	83(70.9)		35(14.8–55.2)		1		49(27.3–70.7)		1		31(20.1–41.9)		1	
TT	33(28.2)		40(0-80.6)		0.992(0.579-1.700)	0.976	65(24.2-105.8)		0.997(0.580–1.716)	0.992	45(21.8–68.2)		0.864(0.539–1.384)	0.543
**Co-dominant model**														
rs4711998				0.960				0.976				0.983		
AA	6 (5.1%)		24(2.5–45.5)		1		32(29.6–34.1)		1		31(10.6–51.4)		1	
AG	44 (37.6%)		35(8.4–61.6)		1.239(0.374–4.107)	0.725	51(15.5–86.5)		1.131(0.339–3.771)	0.841	31(3.8–58.2)		0.930(0.362–2.389)	0.880
GG	67 (57.3%)		41(13.9–66.1)		1.034(0.318–3.365)	0.956	49(4.8–93.2)		1.089(0.335–3.544)	0.887	39(23.7–54.3)		0.918(0.366–2.305)	0.855
rs7708392				0.351				0.823				0.503		
GG	62 (53.0%)		31(13.6–48.4)		1		59(46.2–72.5)		1		38(27.1–48.9)		1	
GC	45 (38.5%)		67(12.8-121.2)		0.716(0.409–1.252)	0.241	84(0-235.9)		0.853(0.487–1.492)	0.577	28(16.8–39.2)		1.141(0.728–1.787)	0.565
CC	9 (7.7%)		55(0-140.9)		0.616(0.241–1.572)	0.310	50(24.0–76.0)		0.843(0.351–2.024)	0.702	84(0-235.9)		0.705(0.316–1.576)	0.395
rs822336				0.504				0.655				0.619		
GG	38 (32.5%)		31(15.6–46.4)		1		49(22.2–75.8)		1		32(12.3–51.7)		1	
GC	50 (42.7%)		67(5.2-128.8)		0.758(0.430–1.334)	0.336	45(0-94.7)		0.889(0.499–1.583)	0.688	32(17.8–46.2)		0.796(0.486–1.304)	0.365
CC	29 (24.8%)		52(12.4–82.6)		0.715(0.369–1.383)	0.319	63(39.1–86.9)		0.842(0.428–1.657)	0.619	49(21.8–76.2)		0.964(0.558–1.663)	0.894
rs10965215				0.726				0.349				0.944		
GG	35 (29.9%)		67(13.1-120.9)		1		51.0(0-120.7)		1		27(0-59.7)		1	
GA	55 (47.0%)		29(16.2–41.8)		1.222(0.677–2.205)	0.506	32.0(12.0–52.0)		1.103(0.613–1.987)	0.743	54(39.5–68.5)		1.029(0.625–1.696)	0.910
AA	27 (23.1%)		40(21.3–58.7)		1(0.492–2.034)	1	65(36.9–93.1)		0.795(0.388–1.628)	0.531	33(20.2–45.8)		0.941(0.532–1.664)	0.834
rs6505162				0.965				0.929				0.745		
AA	38 (32.5%)		53(25.7–78.3)		1		51(24.9–77.1)		1		38(13.6–62.4)		1	
AC	58 (49.6%)		37(14.6–59.4)		1.074(0.610–1.891)	0.804	50(20.1–79.8)		0.971(0.551–1.712)	0.919	41(19.9–62.1)		0.984(0.609–1.588)	0.947
CC	21 (17.9%)		29(19.8–38.2)		1.016(0.483–2.138)	0.966	32(18.9–45.0)		1.111(0.532–2.320)	0.780	28(11.5–44.4)		1.215(0.665–2.222)	0.526
rs7315438	21 (17.9%)			0.726				0.961				0.491		
CC	62 (53.0%)		39(17.0-60.9)		1		49(24.0–74.0)		1		49(23.8–74.2)		1	
CT	33 (28.2%)		31(0-64.1)		0.817(0.441–1.513)	0.520	54(18.6–89.4)		0.912(0.479–1.735)	0.778	28(15.3–40.7)		1.334(0.754–2.360)	0.323
TT	21 (17.9%)		40(0-80.6)		0.868(0.446–1.689)	0.678	65(24.2-105.8)		0.937(0.470–1.870)	0.854	45(21.8–68.2)		1.060(0.563–1.998)	0.856
**Dominant model**														
rs4711998				0.857				0.865				0.453		
AA	6 (5.1%)		24(2.5–45.5)		1		32(29.9–34.1)		1		11(3.3–18.7)		1	
AG/GG	111 (94.9%)		37(23.1–50.9)		1.111(0.348–3.545)	0.859	50(28.2–71.8)		1.105(0.346–3.531)	0.866	27(20.2–33.8)		0.923(0.374–2.278)	0.861
rs7708392				0.155				0.533				0.115		
GG	62 (53.0%)		31(13.6–48.4)		1		50(26.5–73.5)		1		30(16.3–43.7)		1	
GC/CC	54 (46.2%)		67(4.8-129.2)		0.691(0.413–1.158)	0.161	49(0-105.0)		0.850(0.509–1.421)	0.536	21(16.2–25.9)		1.030(0.674–1.574)	0.891
rs822336				0.246				0.609				0.980		
GG	38 (32.5%)		31(15.6–46.4)		1		49.0(22.2–75.8)		1		27(17.8–36.2)		1	
GC/CC	79 (67.5%)		52(10.9–93.1)		0.741(0.444–1.237)	0.252	54(21.2–86.8)		0.872(0.513–1480)	0.611	24(14.3–33.7)		0.856(0.549–1.335)	0.494
rs10965215				0.632				0.965				0.494		
GG	35 (29.9%)		67(13.1-120.9)		1		51.0(0-120.7)		1		25(18.3–31.7)		1	
GA/AA	82 (70.1%)		31(15.3–46.7)		1.145(0.655–2.001)	0.635	49(25.6–72.4)		0.988(0.568–1.719)	0.965	28(17.5–38.6)		0.995(0.627–1.580)	0.985
rs6505162				0.833				0.985				0.029		
AA	38 (32.5%)		52.0(25.7–78.3)		1		51.0(24.9–77.1)		1		41(8.1–73.9)		1	
AC/CC	79 (67.5%)		29.0(11.9–46.1)		1.059(0.618–1.816)	0.835	49(14.6–83.4)		1.005(0.588–1.717)	0.986	22(17.2–26.8)		1.041(0.663–1.634)	0.861
rs7315438				0.536				0.789				0.784		
CC	21 (17.9%)		39(17.0–70.0)		1		49(24.0–74.0)		1		28(20.2–35.8)		1	
CT/TT	95 (81.2%)		37(11.7–62.2)		0.837(0.474–1.478)	0.540	54(22.0–86.0)		0.922(0.507–1.676)	0.790	25(17.4–32.6)		1.225(0.711–2.112)	0.465

The following SNPs were analyzed: IL17A rs4711998, TNIP1 rs7708392, CD274 rs822336, CDKN2B-AS1 rs10965215, NSRP1 rs6505162 and LOC105370003 rs7315438. CD274, CD274 Molecule; CDKN2B-AS1, Cyclin-dependentkinase inhibitor 2B antisense RNA 1; IL17A, Interleukin 17 A; LOC105370003, Predicted long non-coding RNA LOC105370003; NSRP1, Nuclear Speckle Splicing Regulatory Protein 1; TNIP1, TNFAIP3 Interacting Protein1. *, Kaplan–Meier survival analysis;#, univariate Cox regression

### Associations Between Patient Characteristics, Single Nucleotide Polymorphisms and Outcomes in Perihilar Cholangiocarcinoma

Univariate Cox regression models did not identify any SNPs as significant prognostic factors for RFS, CSS, or OS (Table 4). Likewise, multivariate Cox regression (Table 5) showed that none of the genotypes were independently associated with postoperative outcomes in pCCA. Clinical factors such as MVI, nodal stage, and postoperative complications had a more pronounced impact on survival (Table 5). A complete univariate analysis including all clinical variables and SNP data is presented in supplementary table S6.

**Table 5:** Multivariable Cox regression analysis of recurrence-free, cancer-specific and overall survival in perihilar cholangiocarcinoma

Variables	Recurrence-free survival		Cancer-specific survival		Overall survival	
	HR (95% CI)	*p* value	HR( 95% CI)	*p* value	HR (95% CI)	*p* value
Hemoglobin, g/L (≤ 12 = 1)	0.274 (0.146–0.516)	<0.001	0.347(0.180–0.668)	0.002	0.385(0.226–0.654)	< 0.001
Intraoperative FFP (No = 1)	2.868(1.501–5.480)	0.001	4.340(2.094–8.994)	< 0.001	3.072(1.762–3.354)	< 0.001
MVI (No = 1)	48.237(8.791-264.662)	<0.001	50.516(8.685-293.835)	< 0.001	19.083(3.880-93.844)	< 0.001
Tumor grading (G1/G2 = 1)	3.740(1.711–8.177)	<0.001	4.637(2.109–10.193)	< 0.001	3.699(1.879–7.284)	< 0.001
pT category (pT1-2 = 1)	----	--	2.573(1.358–4.877)	0.004	2.227(1.309–3.789)	0.003
N category (pN0 = 1)	2.704(1.476–4.954)	0.001	2.524(1.374–4.634)	0.003	1.779(1.055–2.998)	0.031
Perioperative complications (Clavien-Dindo) (0/I/II = 1)	----	--	----	--	2.115(1.279–3.497)	0.004

Only significant parameters are shown. FFP, fresh frozen plasma; MVI, microvascular invasion

## Discussion

Our study explored the prognostic value of selected SNPs in genes related to inflammation and RNA regulation in cholangiocarcinoma subtypes. Although we identified TNIP1 rs7708392 and NSRP1 rs6505162 as potential prognostic markers in iCCA in terms of OS, these associations were limited by small subgroup sizes in the specific genotypes, notably the very low number of cases carrying TNIP1 rs7708392 CC genotype. Given the lack of consistent associations with oncogically more precise parameters e.g. RFS or CSS, our findings could represent incidental observations rather than clinically relevant biomarkers. Importantly, none of the SNPs studied showed prognostic value in pCCA, underscoring the limited impact of individual SNPs on survival outcomes in CCA.

The concept that inflammation-related and RNA-processing genes can influence cancer outcomes is biologically plausible and supported by prior research. For instance, TNIP1, a key component in autoimmunity and chronic inflammation, encodes an A20-binding protein implicated in nuclear factor k-light-chain-enhancer of activated B cell (NF-κB) regulation and multiple receptor-mediated signaling cascades [18–21]. Rs7708392 in particular is known to affect alternative splicing reulting in the production of protein isoforms with distinct biological characteristics, each possessing unique protein interactions and catalytic properties [22, 23]. From an epidemiological perspective, the G allele of rs7708392 has already been shown to significantly increase hepatocellular carcinoma (HCC) risk in a Northwest Chinese Han population [24]. Likewise, NSRP1 rs6505162 which resides within the pre-miR‑423 region is involved in RNA processing. Prior studies have linked this polymorphism to cancer susceptibility in diverse populations, suggesting that the rs6505162 variant affects the maturation of miR-423 or the splicing of NSRP1 itself, thereby altering expression of genes crucial for cell cycle or apoptosis [25, 26] A recent meta-analyze demonstrates that the AA genotype of rs6505162 is associated with a reduced overall cancer risk showing the most pronounced effect in lung cancer [25]. Another meta-analysis with 5,891 cases and 7,622 controls found that AA/CA genotypes decreased breast cancer risk but increased lung cancer risk, especially in Caucasians [27].

Minor signals for iCCA in our cohort were observed for TNIP1 rs7708392 and NSRP1 rs6505162. The nexus between inflammation and carcinogenesis is well-documented, as genes governing inflammatory responses profoundly influence the cancer trajectory [15]. This particularly applies to SNPs in interleukin genes, including IL4 rs2243250 and IL17A rs4711998, which unfortunately showed no prognostic associations in either iCCA or pCCA within our study. In other entities, research led by Arancha and colleagues elucidated a correlation between the IL4 rs2243250 polymorphism and CSS in metastatic renal cell carcinoma, while other studies have established links between polymorphisms in the IL-17 gene and various cancers, including HCC, and CRC [28–31]. This findings are speculated to be based on functional consequences of the variants: IL4 rs2243250 enhances IL-4 transcription, thereby promoting a Th2-skewed immune response and facilitating tumor immune evasion, whereas IL17A polymorphisms can augment pro-inflammatory signaling and angiogenesis, processes that sustain tumor growth and metastasis [32–34].

This discrepancy is in line with a broader picture emerging from genetic prognostic studies in rare malignancies: even variants with plausible biological functions may exert effects too subtle to be detected in even in relatively large cohorts, or may only influence disease in concert with other genetic and environmental factors [35–37]. For instance, the IL4 rs2243250 variant has been reported to influence cancer susceptibility predominantly among smokers, pointing to a synergistic interaction between tobacco-derived carcinogens and genetically determined immune responses [38]. Likewise, polymorphisms within inflammatory or DNA repair pathways may manifest prognostic effects only in the presence of persistent viral infections. A notable example is the increased risk of hepatocellular carcinoma conferred by specific alleles in individuals chronically infected with hepatitis B virus, whereas no such association is observed in uninfected populations [39]. These findings reinforce the paradigm that genetic predisposition often requires permissive environmental triggers—such as smoking, viral infection, or metabolic stress—to translate into clinically evident outcomes [40]. Accordingly, the prognostic value of many SNPs may remain obscured in unstratified cohorts, underscoring the necessity of integrative study designs that explicitly account for gene–environment interactions [35].

The lack of robust, reproducible associations in our data reinforces the need for caution when interpreting SNP–outcome correlations in small subgroups. Rare genotype categories, as in the case of TNIP1 rs7708392 CC carriers, can yield extreme hazard ratios purely by chance. Without validation in multiple independent datasets such findings risk being overinterpreted. Thus, in our view, none of the nine SNPs examined in this study can be considered reliable prognostic markers for CCA at present.

Nevertheless, our study has important methodological strengths. It represents one large single-centre cohort of patients with a rare disease characterized by well-defined inclusion criteria, rigorous pathological review and long-term follow-up. This design minimizes heterogeneity and ensures robust clinical annotation, thereby providing a solid foundation for translational analyses. The validity of our cohort has been demonstrated by previous work which identified prognostic SNP variants in other areas than RNA-related SNP and inflammation within the same clinical framework [16]. These findings illustrate that our cohort and analytic strategy are capable of uncovering clinically meaningful genetic associations in CCA.

Moreover, investigating SNPs in CCA remains of paramount importance. Cholangiocarcinoma is associated with dismal survival rates despite surgical resection, and there is an urgent need for biomarkers that enable better preoperative risk stratification [41, 42]. SNPs, being stable, cost-effective, and assayable in routine blood samples, offer an attractive tool to guide the selection of surgical candidates and potentially inform postoperative surveillance strategies. As such, while our present analysis did not identify reproducible prognostic variants, our findings underscore both the challenges and the continued relevance of pursuing SNP-based biomarkers in this lethal malignancy.

Several limitations of our study should be acknowledged. First, although our cohort is relatively large for a single-center CCA series, the sample size remains modest when stratified by anatomical subtype and genotype, which reduces statistical power and increases the likelihood of spurious associations in rare variant groups. Further, prevalence of germline SNPs may vary across different geographic and ethnic populations, potentially limiting the generalizability of our findings. Second, we restricted our analysis to a candidate set of nine SNPs, thereby not capturing the broader genetic landscape; genome-wide approaches might reveal additional prognostic variants. Finally, the retrospective design and lack of external validation limit the generalizability of our findings. Prospective, multi-center collaborations will be required to validate and extend these observations.

In conclusion, our findings do not support the clinical utility of the studied inflammation- and RNA-related SNPs as prognostic biomarkers for CCA at present. Future research should employ large-scale collaborative cohorts, possibly incorporating genome-wide approaches, to fully elucidate the genetic underpinnings of cholangiocarcinoma prognosis.

## Conclusion

In conclusion, this study evaluated the prognostic relevance of selected inflammation- and RNA-related single-nucleotide polymorphisms in patients undergoing curative-intent resection for cholangiocarcinoma. Overall, no robust or consistent associations were identified between the investigated SNPs and postoperative outcomes, particularly recurrence-free survival and cancer-specific survival. Although TNIP1 rs7708392 and NSRP1 rs6505162 showed limited associations with overall survival in the intrahepatic cholangiocarcinoma cohort, these findings should be interpreted cautiously because of small genotype subgroup sizes and the absence of consistent effects across clinically relevant endpoints. No prognostic impact of the analyzed SNPs was observed in patients with perihilar cholangiocarcinoma. These results suggest that the investigated inflammation- and RNA-related SNPs are unlikely to serve as reliable standalone prognostic biomarkers in resected cholangiocarcinoma. Larger, multicenter studies with independent validation cohorts are required to clarify the potential role of germline genetic variation in cholangiocarcinoma risk stratification and personalized postoperative management.

## Supplementary Information

Below is the link to the electronic supplementary material.

Supplementary Material 1 (DOCX 44.7 KB)

Supplementary Material 2 (DOCX 19.5 KB)

Supplementary Material 3 (DOCX 23.9 KB)

Supplementary Material 4 (DOCX 24.9 KB)

Supplementary Material 5 (DOCX 20.1 KB)

Supplementary Material 6 (DOCX 21.1 KB)

## Data Availability

No datasets were generated or analysed during the current study.
