# Supplementary material for: Inflammation and RNA-Related Polymorphisms in Resected Cholangiocarcinoma: Prognostic Associations in Intrahepatic and Perihilar Tumors
Source: J Gastrointest Cancer. 2026 Jul 8;57(1):148. doi: 10.1007/s12029-026-01520-z (PMC13346121; doi:10.1007/s12029-026-01520-z)
Supplement: Supplementary file 1 — Supplementary Material 1 (DOCX 44.7 KB) [file 12029_2026_1520_MOESM1_ESM.docx]

**S1 Table. Assessed single nucleoid polymorphisms and their function**

| SNP | Gene | Function | Related Cancer Pathways | Supporting References |
| --- | --- | --- | --- | --- |
| rs2243250 | IL4 | Promoter variant (-590 C/T), influences IL-4 transcription and secretion, shifts Th2 immune response, modulating inflammation-cancer axis | Th2 cytokine signaling, JAK/STAT, immune microenvironment remodeling | [1-3] |
| rs4711998 | IL17A | Affects IL-17A expression/activity, involved in tumor-related inflammation and immune evasion | Th17/IL-17 → NF-κB/MAPK, tumor inflammatory microenvironment | [4, 5] |
| rs7708392 | TNIP1 | Encodes ABIN-1, an A20(TNFAIP3) binding inhibitor; negatively regulates NF-κB, associated with autoimmune/inflammatory disorders, linking inflammation to cancer | NF-κB signaling, innate/adaptive immunity, chronic inflammation-driven carcinogenesis | [6, 7] |
| rs822336 | CD274 | Located in promoter/enhancer region, alters transcription factor binding, regulating PD-L1 expression; linked to immunotherapy response | PD-1/PD-L1 immune checkpoint, tumor immune escape | [8, 9] |
| rs10965215 | CDKN2B-AS1 | lncRNA ANRIL polymorphism affecting transcription/epigenetic regulation of CDKN2A/B-p16/p15 tumor suppressor pathway; associated with multiple cancers | Cell cycle regulation, epigenetic modulation | [10, 11] |
| rs6505162 | NSRP1 | Variant linked to miR-423 expression/maturation, influencing proliferation and apoptosis via post-transcriptional regulation | miRNA–mRNA regulatory axis, apoptosis/proliferation, | [12-14] |
| rs7158663 | MEG3 | lncRNA MEG3 tumor suppressor polymorphism; affects MEG3 expression and its p53-dependent signaling | p53 signaling, epigenetic regulation, apoptosis | [15, 16] |
| rs7315438 | LOC105370003 | Long non-coding RNA locus, limited functional data; reported to increase HCC risk | Potential transcriptional/epigenetic regulation | [17] |
| rs944289 | LncRNA  PTCSC3 | Located at 14q13.3, regulates expression of lincRNA PTCSC3 (tumor suppressor), via altered transcription factor binding; established thyroid cancer susceptibility locus | Thyroid tumorigenesis network (NKX2-1/PTCSC3 axis), differentiation and growth signaling | [18, 19] |

**CD274**, CD274 Molecule; **CDKN2B-AS1**, Cyclin-dependent kinase inhibitor 2B antisense RNA 1; **IL4**, Interleukin 4; **IL17A**, Interleukin 17A; **LncRNA PTCSC3**, Long non-coding RNA of Papillary Thyroid Carcinoma Susceptibility Candidate 3; **LOC105370003**, Predicted long non-coding RNA LOC105370003; **MEG3**, Maternally Expressed Gene 3; **NSRP1**, Nuclear Speckle Splicing Regulatory Protein 1; **TNIP1**, TNFAIP3 Interacting Protein 1.

References:

1. Nakashima H, Miyake K, Inoue Y, Shimizu S, Akahoshi M, Tanaka Y, Otsuka T, Harada M: **Association between IL-4 genotype and IL-4 production in the Japanese population**. *Genes Immun* 2002, **3**(2):107-109.

2. Shamran HA, Hamza SJ, Yaseen NY, Al-Juboory AA, Taub DD, Price RL, Nagarkatti M, Nagarkatti PS, Singh UP: **Impact of single nucleotide polymorphism in IL-4, IL-4R genes and systemic concentration of IL-4 on the incidence of glioma in Iraqi patients**. *Int J Med Sci* 2014, **11**(11):1147-1153.

3. Suzuki A, Leland P, Joshi BH, Puri RK: **Targeting of IL-4 and IL-13 receptors for cancer therapy**. *Cytokine* 2015, **75**(1):79-88.

4. Pawlik A, Kotrych D, Malinowski D, Dziedziejko V, Czerewaty M, Safranow K: **IL17A and IL17F gene polymorphisms in patients with rheumatoid arthritis**. *BMC Musculoskelet Disord* 2016, **17**:208.

5. Amatya N, Garg AV, Gaffen SL: **IL-17 Signaling: The Yin and the Yang**. *Trends Immunol* 2017, **38**(5):310-322.

6. G'Sell RT, Gaffney PM, Powell DW: **A20-Binding Inhibitor of NF-κB Activation 1 is a Physiologic Inhibitor of NF-κB: A Molecular Switch for Inflammation and Autoimmunity**. *Arthritis Rheumatol* 2015, **67**(9):2292-2302.

7. Gurevich I, Zhang C, Francis N, Aneskievich BJ: **TNIP1, a retinoic acid receptor corepressor and A20-binding inhibitor of NF-κB, distributes to both nuclear and cytoplasmic locations**. *J Histochem Cytochem* 2011, **59**(12):1101-1112.

8. Polcaro G, Liguori L, Manzo V, Chianese A, Donadio G, Caputo A, Scognamiglio G, Dell'Annunziata F, Langella M, Corbi G *et al*: **rs822336 binding to C/EBPβ and NFIC modulates induction of PD-L1 expression and predicts anti-PD-1/PD-L1 therapy in advanced NSCLC**. *Mol Cancer* 2024, **23**(1):63.

9. Polcaro G, Liguori L, Manzo V, Chianese A, Donadio G, Caputo A, Scognamiglio G, Dell'Annunziata F, Langella M, Corbi G *et al*: **Abstract 2531: rs822336 as a predictive biomarker for anti-PD-1/PD-L1 immunotherapy in advanced NSCLC: Unraveling molecular mechanisms**. *Cancer research* 2024, **84**(6_Supplement):2531-2531.

10. Drak Alsibai K, Vacher S, Meseure D, Nicolas A, Lae M, Schnitzler A, Chemlali W, Cros J, Longchampt E, Cacheux W *et al*: **High Positive Correlations between ANRIL and p16-CDKN2A/p15-CDKN2B/p14-ARF Gene Cluster Overexpression in Multi-Tumor Types Suggest Deregulated Activation of an ANRIL-ARF Bidirectional Promoter**. *Noncoding RNA* 2019, **5**(3).

11. Liu F, Xiao Y, Ma L, Wang J: **Regulating of cell cycle progression by the lncRNA CDKN2B-AS1/miR-324-5p/ROCK1 axis in laryngeal squamous cell cancer**. *Int J Biol Markers* 2020, **35**(1):47-56.

12. Chen R, Zheng Y, Zhuo L, Wang S: **The association between miR-423 rs6505162 polymorphism and cancer susceptibility: a systematic review and meta-analysis**. *Oncotarget* 2017, **8**(25):40204-40213.

13. Moazeni-Roodi A, Ghavami S, Hashemi M: **Association Between miR-423 rs6505162 Polymorphism and Susceptibility to Cancer**. *Archives of Medical Research* 2019, **50**(1):21-30.

14. Han Z, Jiang G, Zhang Y, Xu J, Chen C, Zhang L, Xu Z, Du X: **Effects of RNA interference-mediated NRP-1 silencing on the proliferation and apoptosis of breast cancer cells**. *Mol Med Rep* 2015, **12**(1):513-519.

15. Gao X, Li X, Zhang S, Wang X: **The Association of MEG3 Gene rs7158663 Polymorphism With Cancer Susceptibility**. *Front Oncol* 2021, **11**:796774.

16. Zhou Y, Zhong Y, Wang Y, Zhang X, Batista DL, Gejman R, Ansell PJ, Zhao J, Weng C, Klibanski A: **Activation of p53 by MEG3 non-coding RNA**. *The Journal of biological chemistry* 2007, **282**(34):24731-24742.

17. Li Y, He HC, Zhou DL, Liu Q, Zhang X, Yang XH, Ye ZL, Peng JL, Tang T, Su X *et al*: **Associations between lncRNA-related polymorphisms and hepatocellular carcinoma risk: A two-stage case-control study**. *J Gastroenterol Hepatol* 2021, **36**(1):233-239.

18. Jendrzejewski J, He H, Radomska HS, Li W, Tomsic J, Liyanarachchi S, Davuluri RV, Nagy R, de la Chapelle A: **The polymorphism rs944289 predisposes to papillary thyroid carcinoma through a large intergenic noncoding RNA gene of tumor suppressor type**. *Proc Natl Acad Sci U S A* 2012, **109**(22):8646-8651.

19. Jendrzejewski J, Thomas A, Liyanarachchi S, Eiterman A, Tomsic J, He H, Radomska HS, Li W, Nagy R, Sworczak K *et al*: **PTCSC3 Is Involved in Papillary Thyroid Carcinoma Development by Modulating S100A4 Gene Expression**. *J Clin Endocrinol Metab* 2015, **100**(10):E1370-1377.
