# Supplementary material for: Inflammation and RNA-Related Polymorphisms in Resected Cholangiocarcinoma: Prognostic Associations in Intrahepatic and Perihilar Tumors
Source: J Gastrointest Cancer. 2026 Jul 8;57(1):148. doi: 10.1007/s12029-026-01520-z (PMC13346121; doi:10.1007/s12029-026-01520-z)
Supplement: Supplementary file 2 — Supplementary Material 2 (DOCX 19.5 KB) [file 12029_2026_1520_MOESM2_ESM.docx]

**S2 Table. Genes and their single nucleotide polymorphism sequences**

| **SNP** | **Gene** | **Location** | **Context Sequence[VIC/FAM]** |  |
| --- | --- | --- | --- | --- |
| rs2243250 | *IL4* | Chr.5: 132673462 | ACACCTAAACTTGGGAGAACATTGT[C/T]CCCCAGTGCTGGGGTAGGAGAGTCT |  |
| rs4711998 | *IL17A* | Chr.6: 52185555 | GTATTCCTGAGAAGGAACTATTCTC[A/G]AGGACCTGAGTCCAAGTTCATCTTA |  |
| rs7708392 | *TNIP1* | Chr.5: 151077924 | CGAGGAGAGGCTGATTCCAGTTATT[G/C]TGACTAGTCTACTAAGTTCCAGAAG |  |
| rs822336 | *CD274* | Chr.9: 5448690 | TTACTAATACGCAAATCACTGAGCA[C/G]CAAGCTGAGCAAATACCCTCAATTC |  |
| *rs10965215* | *CDKN2B-AS1* | *Chr.9: 22029446* | *GATGTTTTGCAGGACTATTTGCCAC[A/G]ACATTTCAAAGGATTCCAAGAGAGA* |  |
| *rs6505162* | *NSRP1* | *Chr.17: 30117165* | *TGAGGCCCCTCAGTCTTGCTTCCTA[A/C]CCCGCGCTTGAGTTTCTCCCCGCTT* |  |
| *rs7158663* | *MEG3* | *Chr.14: 100853087* | *ATGGCACAAAAGCCAGAGATAAAAC[A/G]TCCTTCACGTGCTCCCTACCCGGTT* |  |
| *rs7315438* | | *LOC105370003* | *Chr.12: 115453598* | *GGGCAAAGCCACTGGGCCAAGAAGG[C/T]CAACAAGGTGGTGCCAAAAGCACAT* |
| *rs944289* | *LncRNA*  *PTCSC3* | *Chr.14: 36180040* | *TAATTTGGTTGAAAGATAGTCATTG[C/T]AGATTTGTAATAGCTGGGAATTTAA* |  |

**CD274**, CD274 Molecule; **CDKN2B-AS1**, Cyclin-dependent kinase inhibitor 2B antisense RNA 1; **IL4**, Interleukin 4; **IL17A**, Interleukin 17A; **LncRNA PTCSC3**, Long non-coding RNA of Papillary Thyroid Carcinoma Susceptibility Candidate 3; **LOC105370003**, Predicted long non-coding RNA LOC105370003; **MEG3**, Maternally Expressed Gene 3; **NSRP1**, Nuclear Speckle Splicing Regulatory Protein 1; **TNIP1**, TNFAIP3 Interacting Protein 1.
