# Supplementary material for: Inflammation and RNA-Related Polymorphisms in Resected Cholangiocarcinoma: Prognostic Associations in Intrahepatic and Perihilar Tumors
Source: J Gastrointest Cancer. 2026 Jul 8;57(1):148. doi: 10.1007/s12029-026-01520-z (PMC13346121; doi:10.1007/s12029-026-01520-z)
Supplement: Supplementary file 3 — Supplementary Material 3 (DOCX 23.9 KB) [file 12029_2026_1520_MOESM3_ESM.docx]

**S3 Table. Polymorphisms, genotypes, allele frequencies and Hardy-Weinberg Equilibrium in intrahepatic cholangiocarcinoma.**

| iCCA | ID | Gene | *Genotype* | | | *Allele* | | | *HWE p-value* |
| --- | --- | --- | --- | --- | --- | --- | --- | --- | --- |
|  |  |  | *G* | n | % | *A* | n | % | *p* |
| Inflammation | rs2243250 | *IL4* | *CC* | 76 | 67.9 | C | 183 | 81.7 | *0.62* |
|  |  |  | *CT* | 31 | 27.7 | T | 41 | 18.3 |  |
|  |  |  | *TT* | 5 | 4.5 |  |  |  |  |
|  | rs4711998 | *IL17A* | *AA* | 7 | 6.3 | A | 44 | 19.6 | *0.11* |
|  |  |  | *AG* | 30 | 26.8 | G | 180 | 80.4 |  |
|  |  |  | *GG* | 75 | 67.0 |  |  |  |  |
|  | rs7708392 | *TNIP1* | *GG* | 63 | 56.3 | G | 171 | 76.3 | *0.24* |
|  |  |  | *GC* | 45 | 40.2 | C | 53 | 23.7 |  |
|  |  |  | *CC* | 4 | 3.6 |  |  |  |  |
|  | rs822336 | *CD274* | *GG* | 29 | 25.9 | G | 106 | 47.3 | *0.14* |
|  |  |  | *GC* | 48 | 42.9 | C | 118 | 52.7 |  |
|  |  |  | *CC* | 35 | 31.3 |  |  |  |  |
| RNA | *rs10965215* | CDKN2B-AS1 | *GG* | 32 | 28.6 | G | 114 | 50.89 | *0.26* |
|  |  |  | GA | 50 | 44.6 | A | 110 | 49.11 |  |
|  |  |  | AA | 30 | 26.8 |  |  |  |  |
|  | *rs6505162* | NSRP1 | *AA* | 30 | 26.8 | A | 119 | 53.13 | *0.37* |
|  |  |  | *AC* | 59 | 49.6 | C | 105 | 46.88 |  |
|  |  |  | *CC* | 23 | 20.5 |  |  |  |  |
|  | rs7158663 | MEG3 | *AA* | 32 | 28.6 | A | 107 | **48.20** | ***0.02*** |
|  |  |  | *AG* | 43 | 38.4 | G | 115 | 51.80 |  |
|  |  |  | *GG* | 36 | 32.1 |  |  |  |  |
|  | *rs7315438* | LOC105370003 | *CC* | 16 | 14.3 | C | 81 | 36.16 | *0.58* |
|  |  |  | *CT* | 49 | 43.8 | T | 143 | 63.84 |  |
|  |  |  | *TT* | 47 | 42.0 |  |  |  |  |
|  | *rs944289* | *LncRNA*  *PTCSC3* | *CC* | 17 | 15.2 | C | 71 | 34.13 | ***0.03*** |
|  |  |  | *CT* | 37 | 33.0 | T | 137 | 65.87 |  |
|  |  |  | *TT* | 50 | 44.6 |  |  |  |  |

**CD274**, CD274 Molecule; **CDKN2B-AS1**, Cyclin-dependent kinase inhibitor 2B antisense RNA 1; **HWE**, Hardy Weinberg Equilibrium; **IL4**, Interleukin 4; **IL17A**, Interleukin 17A; **LncRNA PTCSC3**, Long non-coding RNA of Papillary Thyroid Carcinoma Susceptibility Candidate 3; **LOC105370003**, Predicted long non-coding RNA LOC105370003; **MEG3**, Maternally Expressed Gene 3; **NSRP1**, Nuclear Speckle Splicing Regulatory Protein 1; **TNIP1**, TNFAIP3 Interacting Protein 1.
