# Supplementary material for: Inflammation and RNA-Related Polymorphisms in Resected Cholangiocarcinoma: Prognostic Associations in Intrahepatic and Perihilar Tumors
Source: J Gastrointest Cancer. 2026 Jul 8;57(1):148. doi: 10.1007/s12029-026-01520-z (PMC13346121; doi:10.1007/s12029-026-01520-z)
Supplement: Supplementary file 4 — Supplementary Material 4 (DOCX 24.9 KB) [file 12029_2026_1520_MOESM4_ESM.docx]

**S4 Table. Polymorphisms, genotypes, allele frequencies and Hardy-Weinberg Equilibrium in perihilar Cholangiocarcinoma**

| P  CCA | ID | Gene | *Genotype* | | | *Allele* | | | *HWE p-value* |
| --- | --- | --- | --- | --- | --- | --- | --- | --- | --- |
|  |  |  | *G* | n | % | *A* | n | % | *p* |
| Inflammation | *rs2243250* | *IL4* | *CC* | *93* | *79.5* | *C* | *204* | *87.2* | ***0.01*** |
|  |  |  | *CT* | *18* | *15.4* | *T* | *30* | *12.8* |  |
|  |  |  | *TT* | *6* | *5.1* |  |  |  |  |
|  | *rs4711998* | *IL17A* | *AA* | *6* | *5.1* | *A* | *56* | *23.9* | *0.72* |
|  |  |  | *AG* | *44* | *37.6* | *G* | *178* | *76.1* |  |
|  |  |  | *GG* | *67* | *57.3* |  |  |  |  |
|  | *rs7708392* | *TNIP1* | *GG* | *62* | *53.0* | *G* | *163* | *42.8* | *0.83* |
|  |  |  | *GC* | *45* | *38.5* | *C* | *63* | *27.2* |  |
|  |  |  | *CC* | *9* | *7.7* |  |  |  |  |
|  | *rs822336* | *CD274* | *GG* | *38* | *32.5* | *G* | *126* | *53.8* | *0.13* |
|  |  |  | *CG* | *50* | *42.7* | *C* | *108* | *46.2* |  |
|  |  |  | *CC* | *29* | *24.8* |  |  |  |  |
| *RNA* | *rs10965215* | *CDKN2B-AS1* | *GG* | *35* | *29.9* | *G* | *125* | *53.42* | *0.55* |
|  |  |  | *GA* | *55* | *47.0* | *A* | *109* | *46.58* |  |
|  |  |  | *AA* | *27* | *23.1* |  |  |  |  |
|  | *rs6505162* | *NSRP1* | *AA* | *38* | *32.5* | *A* | *134* | *57.26* | *0.89* |
|  |  |  | *AC* | *58* | *49.6* | *C* | *100* | *42.74* |  |
|  |  |  | *CC* | *21* | *17.9* |  |  |  |  |
|  | *rs7158663* | *MEG3* | *AA* | *28* | *23.9* | *A* | *102* | *44.35* | ***0.04*** |
|  |  |  | *AG* | *46* | *39.3* | *G* | *128* | *55.65* |  |
|  |  |  | *GG* | *41* | *35.0* |  |  |  |  |
|  | *rs7315438* | *LOC105370003* | *CC* | *21* | *17.9* | *C* | *104* | *44.83* | *0.39* |
|  |  |  | *CT* | *62* | *53.0* | *T* | *128* | *55.17* |  |
|  |  |  | *TT* | *33* | *28.2* |  |  |  |  |
|  | *rs944289* | *LncRNA*  *PTCSC3* | *CC* | *27* | *23.1* | *C* | *94* | *34.35* | ***0.01*** |
|  |  |  | *CT* | *40* | *34.2* | *T* | *130* | *65.65* |  |
|  |  |  | *TT* | 45 | 38.5 |  |  |  |  |

**CD274**, CD274 Molecule; **CDKN2B-AS1**, Cyclin-dependent kinase inhibitor 2B antisense RNA 1; **HWE**, Hardy Weinberg Equilibrium; **IL4**, Interleukin 4; **IL17A**, Interleukin 17A; **LncRNA PTCSC3**, Long non-coding RNA of Papillary Thyroid Carcinoma Susceptibility Candidate 3; **LOC105370003**, Predicted long non-coding RNA LOC105370003; **MEG3**, Maternally Expressed Gene 3; **NSRP1**, Nuclear Speckle Splicing Regulatory Protein 1; **TNIP1**, TNFAIP3 Interacting Protein 1.
