# Supplementary material for: Inflammation and RNA-Related Polymorphisms in Resected Cholangiocarcinoma: Prognostic Associations in Intrahepatic and Perihilar Tumors
Source: J Gastrointest Cancer. 2026 Jul 8;57(1):148. doi: 10.1007/s12029-026-01520-z (PMC13346121; doi:10.1007/s12029-026-01520-z)
Supplement: Supplementary file 5 — Supplementary Material 5 (DOCX 20.1 KB) [file 12029_2026_1520_MOESM5_ESM.docx]

**S5 Table. Patient characteristics in association with recurrence free survival, cancer specific survival and overall survival in intrahepatic cholangiocarcinoma**

| **Variables** | **Recurrence free survival** | | **Cancer specific survival** | | **Overall survival** | |
| --- | --- | --- | --- | --- | --- | --- |
|  | **HR(95% CI)** | **p value** | **HR(95% CI)** | **p value** | **HR(95% CI)** | **p value** |
| Sex(Male=1) | 1.189(0.750-1.883) | 0.462 | 0.825(0.513-1.326) | 0.426 | 0.734(0.475-1.134) | 0.164 |
| Age(≤65=1) | 0.877(0.557-1.380) | 0.570 | 1.043(0.652-1.670) | 0.860 | 1.241(0.804-1.914) | 0.329 |
| BMI(≤25=1) | 0.814(0.519-1.276) | 0.369 | 0.982(0.613-1.574) | 0.940 | 0.988(0.639-1.529) | 0.958 |
| ASA(I/II =1) | 1.071(0.683-1.681) | 0.764 | 1.317(0.820-2.117) | 0.255 | 1.723(1.098-2.705) | **0.018** |
| Cholangitis(No=1) | 0.042(0.001-1.727) | 0.240 | 0.197(0.027-1.425) | 0.108 | 0.465(0.145-1.491) | 0.198 |
| PVE(No=1) | 1.295(0.519-3.234) | 0.580 | 1.271(0.462-3.500) | 0.624 | 1.702(0.737-3.928) | 0.213 |
| Neoadjuvant therapy(No=1) | **2.194(1.043-4.617)** | **0.038** | 1.489(0.643-3.447) | 0.353 | 1.311(0.597-2.877) | 0.499 |
| Albumin, g/dL(≤42=1) | 0.620(0.370-1.040) | 0.135 | 0.529(0.305-0.916) | **0.023** | 0.423(0.252-0.710) | **0.001** |
| AST, U/L(≤40=1) | 1.316(0.825-2.099) | 0.250 | 1.470(0.913-2.367) | 0.113 | 1.343(0.860-2.098) | 0.194 |
| ALT, U/L(≤40=1) | 0.933(0.547-1.591) | 0.800 | 1.319(0.766-2.272) | 0.317 | 1.181(0.710-1.964) | 0.521 |
| GGT, U/L(≤100=1) | **1.542(0.972-2.446)** | **0.066** | 1.678(1.036-2.717) | **0.035** | 1.703(1.083-2.678) | **0.021** |
| Bilirubin, mg/dl(≤1=1) | 0.697(0.335-1.453) | 0.336 | 1.205(0.616-2.359) | 0.585 | 1.227(0.663-2.273) | 0.515 |
| Platelet count,1/nL(≤200=1) | 0.909(0.528-1.563) | 0.729 | 0.782(0.452-1.355) | 0.381 | 0.743(0.446-1.238) | 0.255 |
| AP, U/L(≤100=1) | **1.732(1.069-2.806)** | **0.026** | **1.804(1.083-3.006)** | **0.023** | 1.738(1.081-2.793) | **0.023** |
| Prothrombin time(≤110=1) | 0.815(0.516-1.287) | 0.381 | 0.614(0.379-0.994) | **0.047** | 0.649(0.414-1.016) | **0.059** |
| INR(≤1=1) | 1.295(0.813-2.063) | 0.277 | 1.473(0.914-2.374) | 0.111 | 1.554(1.001-2.413) | **0.050** |
| Hemoglobin,g/L(≤12=1) | **0.582(0.341-0.995)** | **0.048** | 0.471(0.270-0.823) | **0.008** | 0.454(0.270-0.762) | **0.003** |
| CRP, mg/L(≤10=1) | **1.768(1.098-2.846)** | **0.019** | 2.227(1.370-3.618) | **0.001** | 2.237(1.421-3.522) | **＜0.001** |
| Operative time, min(≤360=1) | 1.434(0.841-2.443) | 0.185 | 1.568(0.911-2.699) | 0.104 | 1.635(0.985-2.713) | **0.057** |
| Intraop PRBC (No =1) | **1.775(1.088-2.896)** | **0.022** | 1.919(1.174-3.136) | **0.009** | 1.758(1.111-2.783) | **0.016** |
| Intraop FFP( No =1) | **1.673(1.040-2.691)** | **0.034** | 1.991(1.237-3.205) | **0.005** | 1.866(1.199-2.905) | **0.006** |
| R status(R0=1) | 1.400(0.814-2.410) | 0.224 | 1.570(0.925-2.664) | 0.105 | 1.418(0.862-2.333) | 0.169 |
| MVI(No=1) | **3.917(1.407-10.908)** | **0.009** | 4.495(1.780-11.352) | **0.001** | 3.690(1.469-9.271) | **0.005** |
| LVI(No=1) | **2.992(1.657-5.404)** | **＜0.001** | 3.268(1.827-5.846) | **＜0.001** | 3.402(1.983-5.839) | **＜0.001** |
| Tumor grading(G1/G2=1) | 1.448(0.858-2.443) | 0.165 | 1.969(1.169-3.317) | 0.145 | 1.885(1.147-3.098) | **0.012** |
| Tumor stage UICC( I/II =1) | **2.193(1.309-3.676)** | **0.003** | 2.624(1.548-4.448) | **＜0.001** | 2.797(1.709-4.579) | **＜0.001** |
| pT category(pT1-2=1) | 1.412(0.760-2.623) | 0.275 | 2.009(1.148-3.515) | **0.015** | 1.936(1.143-3.278) | **0.014** |
| N category( pN0=1) | **2.708(1.627-4.506)** | **＜0.001** | 3.222(1.938-5.358) | **＜0.001** | 3.072(1.911-4.940) | **＜0.001** |
| ICU time,days(≤1=1) | 0.973(0.535-1.772) | 0.930 | 1.295(0.706-2.372) | 0.403 | 1.309(0.744-2.304) | 0.350 |
| Hospitalization,days(≤14=1) | **1.910(1.208-3.020)** | **0.006** | 1.737(1.080-2.792) | **0.023** | 1.698(1.093-2.635) | **0.018** |
| Perioperative complications (Clavien-Dindo)(0/I/II =1) | **1.957(1.234-3.105)** | **0.004** | 1.874(1.155-3.040) | **0.011** | 1.777(1.133-2.786) | **0.012** |
| Adjuvant therapy(No=1) | 1.213(0.769-1.914) | 0.407 | 1.070(0.656-1.745) | 0.786 | 0.880(0.550-1.407) | 0.593 |

Univariate analyses are displayed. AP, alkaline phosphatase; ASA, American Society of Anesthesiologists; AST, aspartate aminotransferase; ALT, alanine aminotransferase; BMI, body mass index; CRP, C-reactive protein; FFP, fresh frozen plasma; GGT, gamma-glutamyl transferase; ICU, intensive care unit; INR, international normalized ratio; LVI, lymphovascular invasion; MVI, microvascular invasion; PRBC, packed red blood cells; PVE, portal vein embolization; R, resection margin; UICC, Union for International Cancer Control.
