# Supplementary material for: Inflammation and RNA-Related Polymorphisms in Resected Cholangiocarcinoma: Prognostic Associations in Intrahepatic and Perihilar Tumors
Source: J Gastrointest Cancer. 2026 Jul 8;57(1):148. doi: 10.1007/s12029-026-01520-z (PMC13346121; doi:10.1007/s12029-026-01520-z)
Supplement: Supplementary file 6 — Supplementary Material 6 (DOCX 21.1 KB) [file 12029_2026_1520_MOESM6_ESM.docx]

**S6 Table. Patient characteristics in association with recurrence free survival, cancer specific survival, and overall survival in perihilar cholangiocarcinoma**

| **Variables** | **Recurrence free survival** | | **Cancer specific survival** | | **Overall survival** | |
| --- | --- | --- | --- | --- | --- | --- |
|  | **HR(95% CI)** | **p value** | **HR(95% CI)** | **p value** | **HR(95% CI)** | **p value** |
| Sex(Male=1) | 1.096(0.649-1.851) | 0.732 | 1.241(0.735-2.095) | 0.419 | 1.291(0.834-1.997) | 0.252 |
| Age(≤65=1) | 0.700(0.427-1.148) | 0.158 | 1.087(0.654-1.807) | 0.748 | 1.332(0.863-2.056) | 0.196 |
| BMI(≤25=1) | 1.366(0.833-2.242) | 0.216 | 1.105(0.669-1.827) | 0.696 | 1.217(0.799-1.855) | 0.360 |
| ASA(I/II =1) | 1.209(0.733-1.996) | 0.457 | 1.413(0.843-2.368) | 0.190 | 1.427(0.925-2.202) | 0.108 |
| Cholangitis(No=1) | 1.482(0.861-2.551) | 0.156 | 1.397(0.810-2.408) | 0.229 | 1.368(0.861-2.173) | 0.184 |
| PVE(No=1) | 0.933(0.533-1.573) | 0.795 | 0.945(0.557-1.603) | 0.833 | 0.889(0.570-1.386) | 0.604 |
| Neoadjuvant therapy(No=1) | 0.792(0.193-3.243) | 0.746 | 0.863(0.210-3.542) | 0.838 | 1.194(0.436-3.265) | 0.730 |
| Albumin, g/dL(≤42=1) | 0.565(0.267-1.192) | 0.134 | 0.606(0.273-1.343) | 0.217 | 0.881(0.493-1.577) | 0.670 |
| AST, U/L(≤40=1) | 1.574(0.931-2.661) | **0.090** | 1.241(0.739-2.084) | 0.414 | 1.047(0.683-1.606) | 0.832 |
| ALT, U/L(≤40=1) | 0.867(0.454-1.653) | 0.664 | 0.809(0.422-1.550) | 0.523 | 0.743(0.431-1.280) | 0.284 |
| GGT, U/L(≤100=1) | 1.187(0.429-3.283) | 0.741 | 2.365(0.576-9.710) | 0.232 | 1.363(0.550-3.374) | 0.504 |
| Bilirubin, mg/dl(≤1=1) | 1.395(0.849-2.293) | 0.189 | 1.168(0.706-1.932) | 0.545 | 1.102(0.723-1.679) | 0.652 |
| Platelet count,1/nL(≤200=1) | 1.651(0.661-4.124) | 0.283 | 0.957(0.435-2.107) | 0.913 | 0.871(0.462-1.643) | 0.671 |
| AP, U/L(≤100=1) | 1.577(0.492-5.052) | 0.443 | 2.351(0.572-9.659) | 0.236 | 1.342(0.542-3.327) | 0.525 |
| Prothrombin time(≤110=1) | 0.771(0.456-1.305) | 0.333 | 0.610(0.348-1.071) | **0.085** | 0.781(0.497-1.230) | 0.286 |
| INR(≤1=1) | 1.606(0.961-2.684) | **0.071** | 1.860(1.089-3.178) | **0.023** | 1.386(0.900-2.134) | 0.139 |
| Hemoglobin,g/L(≤12=1) | 0.430(0.261-0.710) | **＜0.001** | 0.522(0.313-0.869) | **0.012** | 0.487(0.318-0.747) | **＜0.001** |
| CRP, mg/L(≤10=1) | 1.003(0.601-1.673) | 0.992 | 1.014(0.603-1.707) | 0.957 | 1.128(0.731-1.740) | 0.586 |
| Operative time, min(≤360=1) | 2.150(1.139-4.059) | **0.018** | 1.814(0.977-3.367) | **0.059** | 1.331(0.827-2.142) | 0.238 |
| Intraop PRBC (No =1) | 1.836(1.113-3.028) | **0.017** | 2.186(1.313-3.638) | **0.003** | 2.409(1.564-3.709) | **＜0.001** |
| Intraop FFP( No =1) | 2.032(1.212-3.407) | **0.007** | 2.878(1.639-5.055) | **＜0.001** | 2.506(1.584-3.963) | **＜0.001** |
| R status(R0=1) | 1.331(0.768-2.307) | 0.309 | 1.133(0.630-2.038) | 0.676 | 1.333(0.830-2.142) | 0.234 |
| MVI(No=1) | 4.379(1.053-18.209) | **0.042** | 6.222(1.480-26.154) | **0.013** | 4.247(1.025-17.601) | **0.046** |
| LVI(No=1) | 2.020(1.135-3.595) | **0.017** | 1.870(1.020-3.428) | **0.043** | 2.089(1.277-3.419) | **0.003** |
| Tumor grading(G1/G2=1) | 2.899(1.594-5.272) | **＜0.001** | 3.105(1.706-5.652) | **＜0.001** | 3.090(1.856-5.145) | **＜0.001** |
| Tumor stage UICC( I/II =1) | 1.906(1.101-3.298) | **0.021** | 2.122(1.215-3.705) | **0.008** | 1.612(1.023-2.539) | **0.040** |
| pT category(pT1-2=1) | 1.932(1.155-3.233) | **0.012** | 2.269(1.354-3.802) | **0.002** | 1.881(1.211-2.924) | **0.005** |
| N category( pN0=1) | 2.233(1.353-3.684) | **0.002** | 2.418(1.456-4.017) | **＜0.001** | 1.813(1.181-2.784) | **0.007** |
| ICU time,days(≤1=1) | 1.611(0.960-2.703) | **0.071** | 1.693(1.000-2.868) | **0.050** | 1.690(1.084-2.636) | **0.021** |
| Hospitalization,days(≤14=1) | 1.137(0.689-1.876) | 0.615 | 1.493(0.888-2.511) | 0.131 | 1.405(0.912-2.165) | 0.123 |
| Perioperative complications (Clavien-Dindo)(0/I/II =1) | 1.580(0.961-2.598) | **0.071** | 1.731(1.045-2.868) | **0.033** | 1.858(1.215-2.841) | **0.004** |
| Adjuvant therapy(No=1) | 1.740(1.015-2.985) | **0.044** | 1.265(0.714-2.240) | 0.421 | 1.246(0.756-2.054) | 0.387 |

Univariate Analyses are displayed. AP, alkaline phosphatase; ASA, American Society of Anesthesiologists; AST, aspartate aminotransferase; ALT, alanine aminotransferase; BMI, body mass index; CRP, C-reactive protein; FFP, fresh frozen plasma; GGT, gamma-glutamyl transferase; ICU, intensive care unit; INR, international normalized ratio; LVI, lymphovascular invasion; MVI, microvascular invasion; PRBC, packed red blood cells; PVE, portal vein embolization; R, resection margin; UICC, Union for International Cancer Control.
